# The Role of Post-translational Modifications on the Energy Landscape of Huntingtin N-Terminus

**DOI:** 10.3389/fmolb.2019.00095

**Published:** 2019-10-01

**Authors:** Havva Yalinca, Charlotte Julie Caroline Gehin, Vladimiras Oleinikovas, Hilal A. Lashuel, Francesco Luigi Gervasio, Annalisa Pastore

**Affiliations:** ^1^Department of Chemistry, University College London, London, United Kingdom; ^2^Laboratory of Molecular and Chemical Biology of Neurodegeneration, Faculty of Life Sciences, Brain Mind Institute, EPFL, Lausanne, Switzerland; ^3^Research Department of Structural and Molecular Biology, University College London, London, United Kingdom; ^4^King's College London, London, United Kingdom

**Keywords:** Huntington's disease, misfolding disease, molecular dynamics, peptide folding, phosphorylation, post-translational modifications, enhanced sampling

## Abstract

Huntington disease is a neurodegenerative disease characterized by a polymorphic tract of polyglutamine repeats in exon 1 of the huntingtin protein, which is thought to be responsible for protein aggregation and neuronal death. The polyglutamine tract is preceded by a 17-residue sequence that is intrinsically disordered. This region is subject to phosphorylation, acetylation and other post-translational modifications *in vivo*, which modulate its secondary structure, aggregation and, subcellular localization. We used Molecular Dynamics simulations with a novel Hamiltonian-replica-exchange-based enhanced sampling method, SWISH, and an optimal combination of water and protein force fields to study the effects of phosphorylation and acetylation as well as cross-talk between these modifications on the huntingtin N-terminus. The simulations, validated by circular dichroism, were used to formulate a mechanism by which the modifications influence helical conformations. Our findings have implications for understanding the structural basis underlying the effect of PTMs in the aggregation and cellular properties of huntingtin.

## Introduction

Huntington disease (HD) is a relentless neurodegenerative disease associated with motor impairments and psychiatric symptoms caused by neurodegeneration of the striatum and the cortex (Nance, 2017). It is caused by the anomalous expansion of CAG trinucleotide repeats within the first exon of the huntingtin gene, which results in a tract of polyglutamine (polyQ) in the gene product, the 348 kDa Huntingtin (Htt) protein. The length of the polyQ tract inversely correlates with the age of disease onset and dictates the severity of the symptoms (Raymond, 2017). PolyQ expansion above 40 residues leads to Htt aggregation in neurons and causes progressive neurodegeneration (Arndt et al., 2015). The polyQ tract is preceded by a lipid-binding N-terminal region of 17 amino acids which has been implicated in interactions with various cellular membranes and regulation Htt cellular properties (Rockabrand et al., 2006; Atwal et al., 2007). This region is subject to phosphorylation, acetylation and other post-translational modifications (PTMs), which influence the aggregation, and subcellular localization of both the full-length protein and N-terminal fragments of Htt (Chaibva et al., 2016; DiGiovanni et al., 2016; Bassi et al., 2017; Cariulo et al., 2017). Because of its important regulatory role in many aspects of Htt structure, aggregation, interactome, toxicity and, cellular properties, the N-terminus has been extensively characterized *in vitro* both in its unmodified form and with several PTMs (Arndt et al., 2015; Chiki et al., 2017; DeGuire et al., 2018). It was shown to be intrinsically disordered in aqueous environments, but to form an amphipathic helix in the presence of 2,2,2-trifluoroethanol (TFE), or dodecyl phosphocholine (DPC) detergent micelles (Michalek et al., 2013). Phosphorylation at Thr3 was proposed to stabilize a helical conformation of Htt(1–19) and reduce the aggregation tendency of the protein in the context of the expanded exon 1 (Chiki et al., 2017). However, the structural basis underlying the helix stabilization effect of T3 phosphorylation and other PTMs remains unknown.

In the present work, we used advanced computational methods validated by experimental evidence on a peptide encompassing Htt(1–19) to study the molecular mechanism of the helix stabilization by phosphorylation and explore the effects of the potential cross-talk between different combinations of PTMs (acetylation and/or phosphorylation) on its helical conformation. The results of the simulations were extensively validated by our previously-published biophysical data (Chiki et al., 2017) and to new circular dichroism experiments.

## Methods

The starting structure used for the Molecular Dynamics simulations was based on the combined solution and solid-state NMR structure of Htt(1–17) in 50% TFE (PDB ID: 2ld0). The dimensions of the simulation box were determined using a fully extended conformation of the Htt(1–19) peptide and a buffer distance of 4 Å (half the cut off distance of van der Waals interactions) between each side of the peptide and the periodic boundary. The resulting box corresponded to a buffer distance of 18 Å. Htt(1–19) was solvated using explicit TIP4PD water molecules (Piana et al., 2015). Any excess charge was neutralized and additional Na+ and Cl- ions were added to a final concentration of 100 mM using Amber LEaP. Amber99SB*-ILDN (Best and Hummer, 2009; Lindorff-Larsen et al., 2010) force field was used with modified residue force fields: T2P and S2P for the phosphorylated threonine and serine residues, respectively (Homeyer et al., 2005), and ALY for acetylated lysine (Khoury et al., 2013).

MD was performed using AMBER (Case et al., 2005). System equilibration consisted of steepest descent energy minimization, temperature (300 K), and pressure equilibration (1 bar) using Langevin thermostat and Berendsen barostat. Positions of protein atoms were restrained during equilibration.

Sampling Water Interfaces through Scaled Hamiltonians (SWISH) (Oleinikovas et al., 2016) enhanced sampling algorithm was used for the production runs, with 6 replicas (1 μs per replica) for each peptide. SWISH is a novel Hamiltonian-replica-exchange-based method which enhances the sampling efficiency by running a number of replicas of the system in which the non-bonded interactions of solvent molecules with the protein are progressively scaled (Oleinikovas et al., 2016). In this study, the Lennard-Jones potential between water and the apolar atoms of the peptide (namely, all C and S atoms and the hydrogen atoms covalently bound to them) were linearly scaled between 0.85 and 1.10, in steps of 0.05, using a scaling coefficient λ. Following the Hamiltonian replica exchange protocol, SWISH simulation periodically attempts swapping adjacent replicas (every 5 ps in the present case) with an acceptance probability determined by a criterion that preserves the correct replica statistics (Sugita and Okamoto, 1999).

A pilot simulation of the wild-type peptide with 8 replicas (with Lennard-Jones potential scaling from 0.6 to 1.3, in steps of 0.1) was run first to determine the optimal scaling range and the gaps between the replicas for the production run.

### Data Analysis

After the simulation, the system was reimaged using CPPTRAJ (Roe and Cheatham, 2013). MDTraj 1.7.2 (McGibbon et al., 2015) was used for DSSP secondary structure assignment and distance calculations. The MD-derived helicity was estimated as a fraction of residues assigned a helical secondary structure (α or 3_10_) averaged over each trajectory. Radius of gyration was calculated using PLUMED driver (Tribello et al., 2014). Hydrogen bonds were defined between two electronegative atoms, one of which is covalently bound to a hydrogen, if the distance between the two heavy atoms is ≤3.5 Å, and the bond angle between the two heavy atoms and the hydrogen (heavy-H-heavy) was > 120**°** (Baker and Hubbard, 1984). Unless otherwise specified, only the unscaled replica statistics (λ = 1.0) are being reported.

## Results and Discussion

Our choice of reference force fields for the peptides, water and ions—i.e., the AMBER99SB*-ILDN (Best and Hummer, 2009; Lindorff-Larsen et al., 2010) force field, the TIP4PD (Piana et al., 2015) model, and CHARMM22 (MacKerell et al., 1998) ions, respectively–was guided by the proven performance of this Amber-type force field in describing the structure and dynamics of protein backbones and side chains with a water model devised to avoid an over-representation of collapsed, compact states (Piana et al., 2015; Kuzmanic et al., 2019). We further improved the conformational sampling of the method by using SWISH Molecular Dynamics. The advantage of this approach on Htt peptides is that not only sampling is enhanced by exchanges among replicas, but also the properties of the peptides are sampled using different water-protein dispersive interactions in the various replicas, reducing the risk of overly compact structures for intrinsically disordered peptides (Best et al., 2014; Piana et al., 2015; Huang et al., 2016). Unlike the original paper, where an opening of hydrophobic cavities was aimed, we applied SWISH scaling in the range of both increased (λ > 1.0), and decreased (λ < 1.0) water interactions. This was designed to accelerate transitions between highly solvated unstructured and hydrophobically collapsed compact states. As a result, this approach provides a more thorough conformational sampling than a standard MD (Supplementary Figures S1, S2). The method paves the way to fine-grained exploration of the effects of water-protein dispersive forces on the compactness of intrinsically disordered peptide (IDP) ensembles.

**Figure 1:**
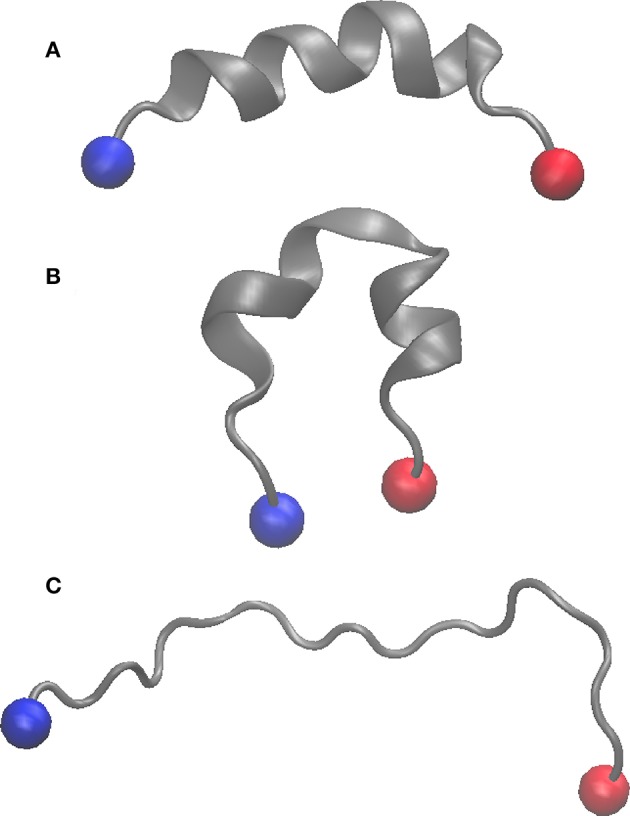
Cartoon representation of the starting structure (PDB ID: 2LD0) **(A)**, replica with lowest lambda value (λ = 0.85) in the WT peptide **(B)**, and that with the highest lambda value (λ = 1.10) **(C)**. The N and C termini are shown as blue and red spheres, respectively.

We first compared the behavior of wild-type (WT) and Thr3 phosphorylated (pThr3) Htt peptides. The starting structure obtained in 50% TFE (PDB ID: 2ld0) contains a continuous helix spanning most of the peptide. This structure equilibrated very rapidly (in approximately 20–50 ns) to a low helical content in water (Supplementary Figure S3). In all our simulations, we observed two approximately helical fragments in agreement with previous MD findings (Binette et al., 2016). Exchange between replicas with different secondary structure content demonstrated that the starting structure does not determine the ensemble that we sample (Figure 1 and Supplementary Figures S1, S2). In excellent agreement with previous circular dichroism (CD) experiments in aqueous solution, we observed that phosphorylation of Thr3 led to a marked increase in helicity on the Htt(1–19) peptide from 6.8 to 11.9%, while additional phosphorylation at Ser13, and/or Ser16 reduced the helical content (Table 1 and Supplementary Figures S4, S5). Phosphorylation of Thr3 affected the structure of the neighboring residues (most notably Lys6, Glu12, Ser13, and Ser16), leading to a different distribution of backbone dihedral angles (Supplementary Figure S6). We also considered the effect of acetylation at the N-terminus and at the ε-amino group of Lys6 in both WT and pThr3 peptides (peptides acMet1, acMet1/pThr3, acLys6, pThr3/acLys6) since we expected the phosphate group to interact with positively charged moieties in its proximity, i.e., the N-terminus and Lys6 (Aurora and Rosee, 1998; Baker et al., 2015).

**Table 1:** Experimental and simulation-derived helical content of the Htt1-19 peptides.

**Modifications**	**Sequence**	**MD derived helicity/%**	**Experimental helicity/%**
WT	MATLEKLMKAFESLKSFQQ	6.8	8
pThr3	MA**pT**LEKLMKAFESLKSFQQ	11.9	15
pThr3, pSer13	MA**pT**LEKLMKAFE**pS**LKSFQQ	8.2	8
pThr3, pSer16	MA**pT**LEKLMKAFESLK**pS**FQQ	9.7	9
pThr3, pSer13, pSer16	MA**pT**LEKLMKAFE**pS**LK**pS**FQQ	8.5	7
pSer16	MATLEKLMKAFESLK**pS**FQQ	7.5	7
pThr3, acLys6	MA**pT**LE**acK**LMKAFESLKSFQQ	6.8	n.d.
acLys6	MATLE**acK**LMKAFESLKSFQQ	12.7	n.d.
pThr3, acMet1	**acM**A**pT**LEKLMKAFESLKSFQQ	14.6	25
acMet1	**acM**ATLEKLMKAFESLKSFQQ	7.0	7
ac2-17 WT	**ac**ATLEKLMKAFESLKSF	n.d.	0.5
ac2-17acLys6	**ac**ATLE**acK**LMKAFESLKSF	n.d.	9

*The modified residues are shown in bold. The indications ac and p stand for acetylation and phosphorylation, respectively. Met1 and Ala2 acetylation is at the N-terminus, while that of Lys6 is on the side chain*.

**Figure 2:**
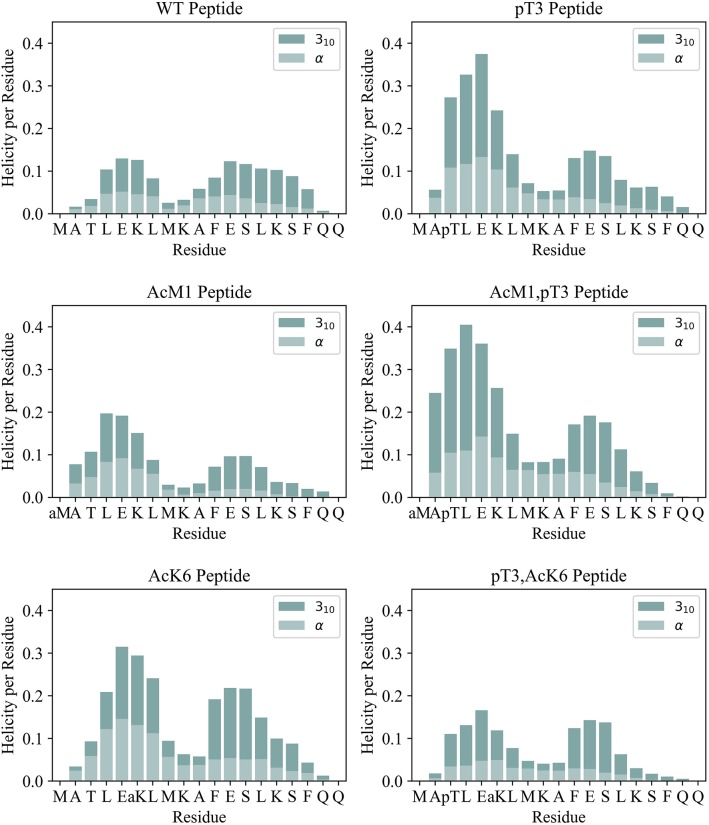
Helicity per residue for the simulated peptides is shown as a stacked bar plot. The helix percentage (divided into α and 3_10_ helix) was evaluated according to DSSP.

**Figure 3:**
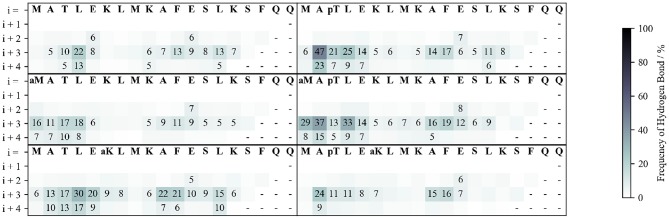
Frequency of backbone hydrogen bond formation. The hydrogen bonds between the carbonyl of each residue (labeled in the 1st row) with the amide proton of each of the following four residues (rows) are shown.

To understand the effect of PTMs on secondary structure, we compared the helical propensity of each residue as evaluated by the DSSP algorithm (Kabsch and Sander, 1983; Figure 2 and Supplementary Figures S7–S9). The secondary structure profiles clearly indicated the presence of two helices and the position of the capping residues, i.e., residues at the helix extremities that stabilize the beginning and termination of the helix. Capping residues typically have helix-like backbone hydrogen bonds but not helical dihedral angles (Aurora and Rosee, 1998). Ala2 acts as the N-terminal helix capping residue (N-cap) in all peptides but WT, where Leu4 is preferred. Phe11 can also be considered as the N-cap of a second helix. This is true for all peptides except for the triple phosphorylated peptide presumably because of a clash between pSer13 and pSer16. These observations are in agreement with the classification of N-capping motifs (Aurora and Rosee, 1998): Ala2 fits the N-capping motif Ib (h-xpxph where h denotes hydrophobic residues or the alkyl side chains of Arg or Lys, p denotes polar residues, x denotes indifference and “–” the helix boundary). Leu4 and Phe11 match the IIa (hp-xpxhx) and Ia (h-xpxhx) motifs, respectively. The first helix in all peptides breaks around residues 8 and 9, leading to a reduction of the helicity in the middle. This is likely due to the repulsion between Lys6 and Lys9. All peptides tend to form preferentially 3_10_ helices rather than α-helices (Figure 3) as expected for isolated peptides in an aqueous solvent (Dyson et al., 1988) but the profiles show interesting distinct features. The WT peptide has comparatively lower helical content compared to the pThr3 peptide where phosphorylation stabilizes a helical conformation in the first half of the peptide, from Ala2 to Leu7. The increase in helical stability of the pThr3 peptide can be attributed in part to the increased negative charge at the helix N-terminus, which counterbalances the helix dipole (Aurora and Rosee, 1998). Acetyl capping of the N-terminus has little effect on the overall helicity of the Htt(1–19) peptide, except for a slight stabilization of the helix closer to the N-terminus in both the phosphorylated and non-phosphorylated forms, implying that the N-terminus is not the major interaction partner of the phosphate group. Acetylation of Lys6 ε-amino group, on the other hand, has a more drastic effect: acetylation at Lys6 alone (without phosphorylation at pThr3) stabilizes the helix to almost the same extent as pThr3 in the MD simulations, while experimental data with similar Htt(2–17) peptides indicate a more modest increase (Table 1). Acetylation of Lys6 in the presence of pThr3 completely abolishes the helix stabilization achieved by phosphorylation at Thr3, resulting in a helicity per residue profile almost identical to that of the WT peptide (Figure 2). The latter observation indicates that the interaction between pThr3 and Lys6 ε-amino group is key in the mechanism of helix stabilization by phosphorylation (Chiki et al., 2017). These findings could partially explain why acetylation at Lys6, but not Lys9, or Lys15 lead to the reversal of the pT3-induced inhibition of mutant Httex1 aggregation.

To understand how these stabilization/destabilization effects correlate with hydrogen bonding and with the secondary structure, we analyzed the backbone and side chain (SC) hydrogen bond patterns and the Ramachandran plots of each peptide along the whole simulation (Figure 3 and Supplementary Figure S10). Compared to WT, pThr3 has a strong tendency to form hydrogen bonds at the N-cap. Phosphorylation of Thr3 leads to a 10-fold increase in the probability of observing a hydrogen bond between Ala2 and Glu5, from 5 to 47%. This dropped to 24% with the introduction of an acetyl group to Lys6 ε-amino group, indicating a link between the pThr3-Lys6 side chain-side chain (SC-SC) salt bridge and Ala2-Glu5 backbone hydrogen bond, with the former potentially playing a role in promoting the overall helicity and leading to H-bonding between Ala2 and Glu5 (Supplementary Table S1). In addition to forming SC-SC salt bridges, the phosphate group also interacts with the peptide backbone and forms hydrogen bonds with the backbone amides of Met1 and Thr3. Acetylation at Lys6 of the pThr3 peptide is accompanied by a decrease in the occurrence of the hydrogen bonds between the pThr3 phosphate oxygens and the backbone amide of pThr3 residue from 54 in pThr3 to 36% in the pThr3/acLys6 peptide (Supplementary Table S2 and Figure 2). The SC-SC hydrogen bond networks of these two peptides, however, are different: the most prominent SC hydrogen bond in the latter is between pThr3 and Lys6. Lys6 is not available as a hydrogen bond donor in the former due to acetylation. Indeed, the side chain-backbone NH hydrogen bond network of the first half of the acLys6 peptide is almost identical to that of WT. This may explain why the helix formed is slightly further downstream as compared to the pThr3 peptide: the N-terminus of the helix in the former lacks the stabilization provided by the hydrogen bonds between the phosphate group and the backbone NH, observed in the latter. An increased helical content in the acLys6 peptide as compared to WT indicates that there may be other mechanisms of helix stabilization in addition to the two key hydrogen bond pairs suggested above, namely pThr3 phosphate-Lys6 amine SC-SC and an Ala2-Glu5 backbone hydrogen bonds.

The overall trend observed in our simulations is in excellent agreement with CD (Table 1) and other experimental data (Chiki et al., 2017; DeGuire et al., 2018). It was for instance previously demonstrated that phosphorylation at Thr3 stabilizes and increases the α-helical conformation in a way which could not be matched by phosphomimetic substitutions (Chiki et al., 2017). Conversely, multiple phosphorylation at residues Thr3, Ser13, and/or Ser16 disrupts the helical conformation of synthetic peptides (DeGuire et al., 2018). Phosphorylation at Ser13, Ser16, or both residues also inhibits aggregation and affects membrane binding. The same modifications of the full-length exon 1 of Htt prompt internalization and nuclear targeting of preformed aggregates (DeGuire et al., 2018). These results suggest that there is a direct correlation between what we observe computationally, the conformational tendencies of the peptides and their tendency to aggregate.

Our results thus provide a direct validation of the SWISH method in intrinsically disordered peptides with a helical tendency. As expected, there are some small differences between experimental and computational data: for example, in our MD simulations, the acLys6 peptide is almost as helical as the pThr3 peptide, with a smaller but significant increased helical content as compared to the unmodified peptide. However, the simulations were run on a peptide containing Met1 whereas the CD data were collected on an acetylated peptide lacking Met1 and thus non-comparable (Table 1).

In conclusion, we provide here a robust simulation-based approach that enabled us to investigate the effect of phosphorylation, and acetylation on the secondary structure of the Htt N-terminus fragment *in silico*. The first approximately 100 residues of Htt, which correspond to exon 1, are intrinsically unfolded when this region is cleaved off from the rest of the protein as it is the case of Huntington's chorea. This means that studying only a fragment is not expected to change the essence of the conclusions. We adapted a state-of-the-art enhanced sampling method used in the context of cryptic pocket formation to simulations of IDPs. Our simulations indicate the mechanism for helix stabilization by phosphorylation, with the contribution of side-chain to backbone hydrogen bonds and effects of charge neutralization by N-terminal acetylation. Thus, we show for the first time that an MD-based method successfully captures the effect of the interplay between key PTMs of a peptide. What is more, we observed an intriguing correlation between the simulation-derived helicity and previous experimental observations of the protective role of phosphorylation and acetylation against aggregation (Chiki et al., 2017; DeGuire et al., 2018). A helical stabilization by acetylation confirmed that aggregation is, at least indirectly, affected by the helical propensity of the peptide and hints at a previously underappreciated role of acetylation. Taken together, the novel mechanistic analysis presented here and the demonstration of a qualitative, or even semi-quantitative, agreement between state-of-the-art simulations, and experiments pave the way to a deeper understanding of the link between PTMs, and the helical content of Htt and other similar peptides.

## Data Availability Statement

All datasets generated for this study are included in the manuscript/Supplementary Files. The raw trajectories are available on the Github page of Gervasio's group at: https://github.com/Gervasiolab/Gervasio-Protein-Dynamics.

## Author Contributions

HY did all the simulations. CG performed CD. VO assisted on the technical aspects of simulations. HL initiated the project. FG supervised the simulations. AP supervised the whole project and finalized the manuscript.

### Conflict of Interest

The authors declare that the research was conducted in the absence of any commercial or financial relationships that could be construed as a potential conflict of interest.
