# Supplementary material for: The Role of Post-translational Modifications on the Energy Landscape of Huntingtin N-Terminus
Source: Front Mol Biosci. 2019 Oct 1;6:95. doi: 10.3389/fmolb.2019.00095 (PMC6779701; doi:10.3389/fmolb.2019.00095)
Supplement: Supplementary file 1 [file Presentation_1.pdf]

Supplementary Information

Table S1: Frequency of side chain to side chain salt bridges in all peptides. Salt bridges are defined between two opposite charged atoms within a distance of 4 Å (or 5 Å in brackets).

Peptide	Frequency of salt bridge between residues / %			
	**pThr3-Lys6**	**pSer16-Lys15**	**pSer13-Lys15**	**pSer13-Lys9**
**WT**	n/a	n/a	n/a	n/a
**pThr3**	21 (29)	n/a	n/a	n/a
**pThr3, pSer13**	29 (36)	n/a	6 (10)	0 (12)
**pThr3, pSer16**	29 (36)	16 (19)	n/a	n/a
**pThr3, pSer13, pSer16**	21 (27)	20 (24)	9 (15)	7 (10)
**pSer16**	n/a	27 ()	n/a	n/a
**pThr3, acLys6**	n/a	n/a	n/a	n/a
**acLys6**	n/a	n/a	n/a	n/a
**pThr3, acMet1**	48 (55)	n/a	n/a	n/a
**acMet1**	n/a	n/a	n/a	n/a

Table S2: Side chains to backbone hydrogen bonds. Frequency of Hydrogen bonds of selected side chains with their own backbone amide proton.

Peptide	Frequency of Hydrogen bond of residue to backbone NH / %		
	**pThr3**	**pSer13**	**pSer16**
**WT**	n/a	n/a	n/a
**pThr3**	54	n/a	n/a
**pThr3, pSer13**	36	0	n/a
**pThr3, pSer16**	40	n/a	0
**pThr3, pSer13, pSer16**	43	0	0
**pSer16**	n/a	n/a	0
**pThr3, acLys6**	36	n/a	n/a
**acLys6**	n/a	n/a	n/a
**pThr3, acMet1**	16	n/a	n/a
**acMet1**	n/a	n/a	n/a

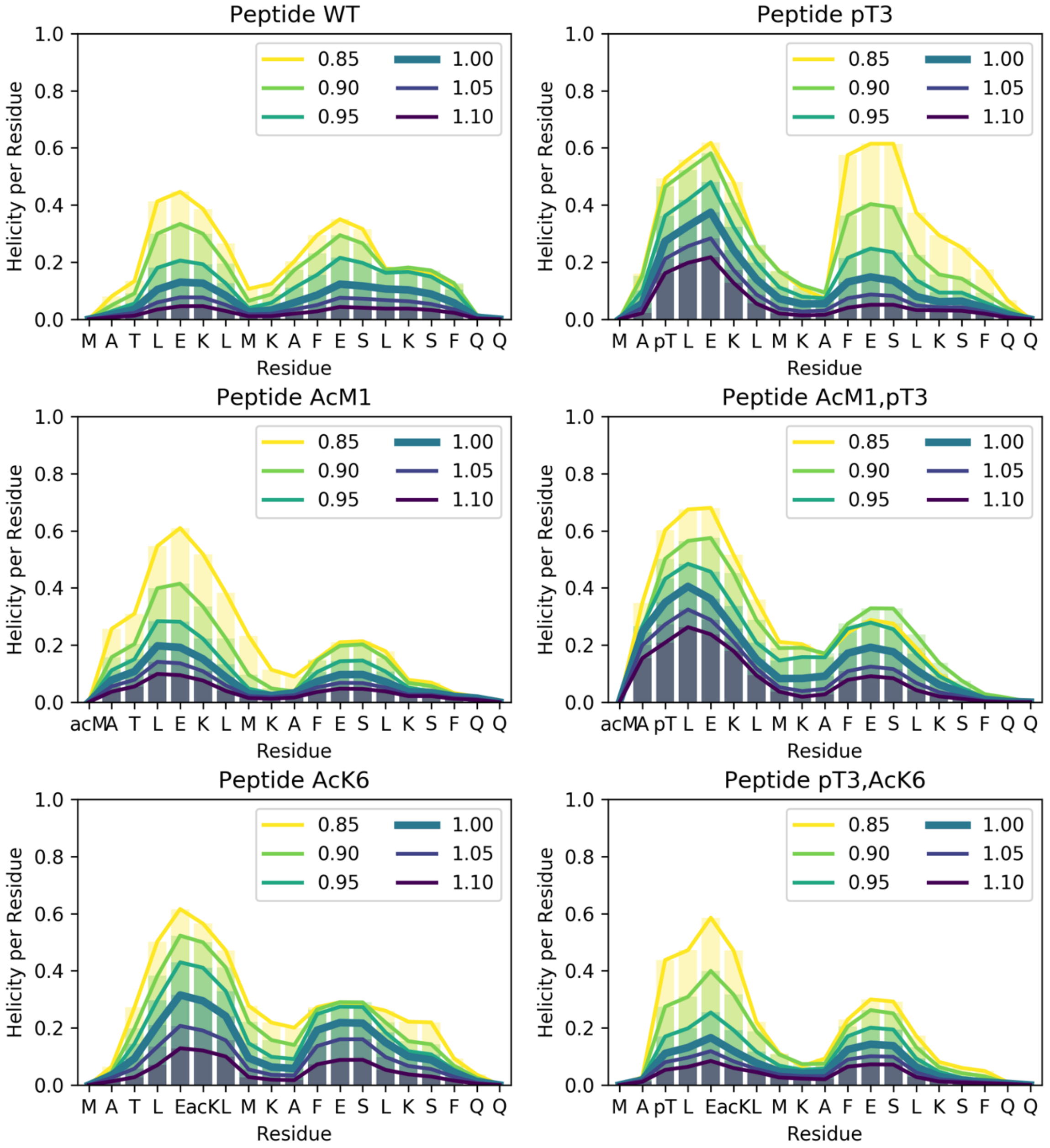

Supplementary Figure S1: Helicity per residue of selected Htt peptides. All 6 replicas in the replica exchange MD are shown, overlapping with one another. Each replica has a different secondary structure content. Thus, exchange between replicas enhances sampling.

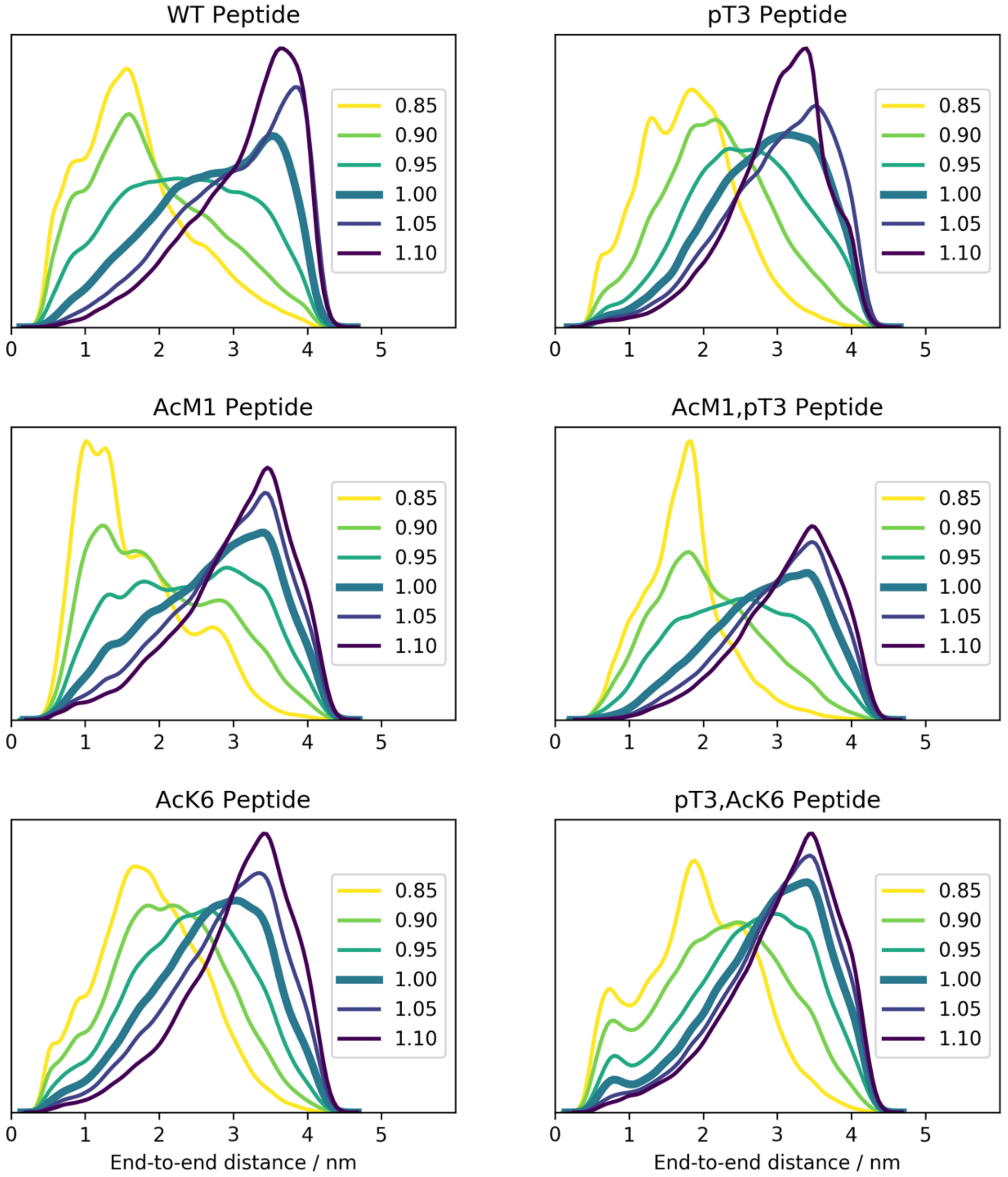

Supplementary Figure S2: End-to-end distance distribution of selected Huntingtin peptides. All 6 replicas in the replica exchange MD are shown. Each replica has a different compactness. Thus, exchange between replicas enhances sampling.

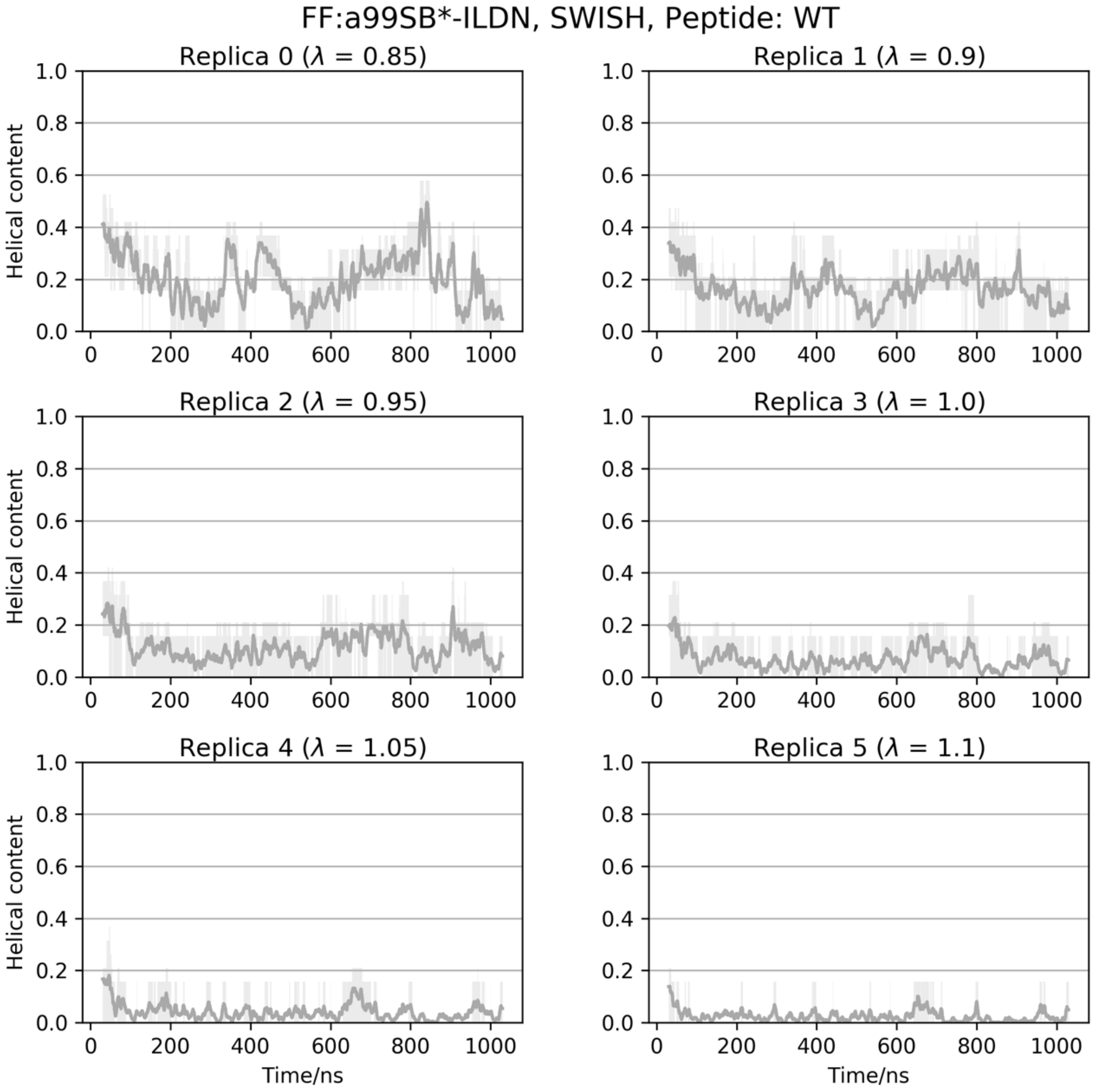

Supplementary Figure S3: Equilibration of the WT peptide from the starting structure in 50% TFE (PDB ID: 2ld0).

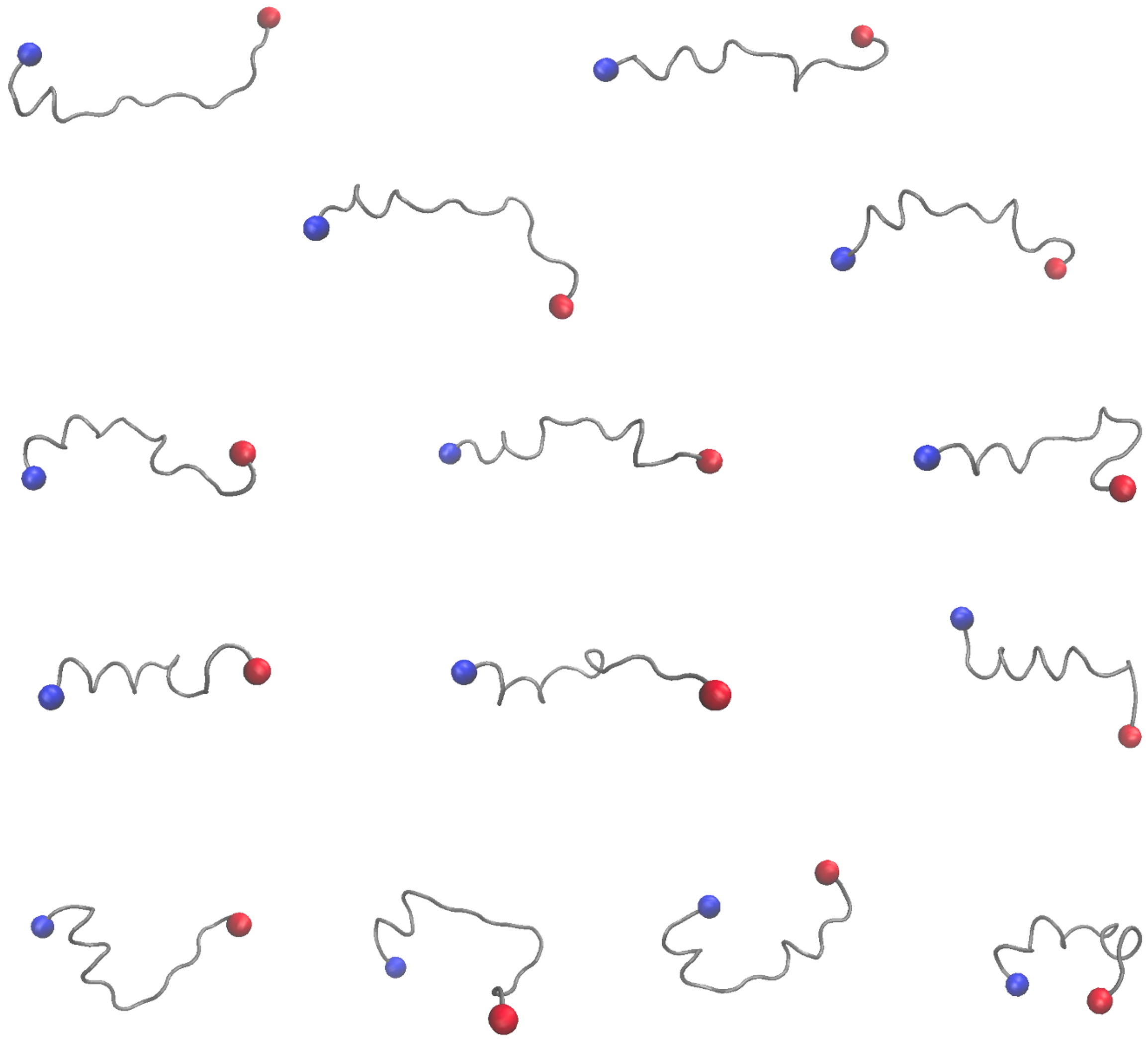

Supplementary Figure S4: Representative structures from pT3 SWISH MD simulation shows stabilisation of a helical conformation near the N terminus of Htt1-19.

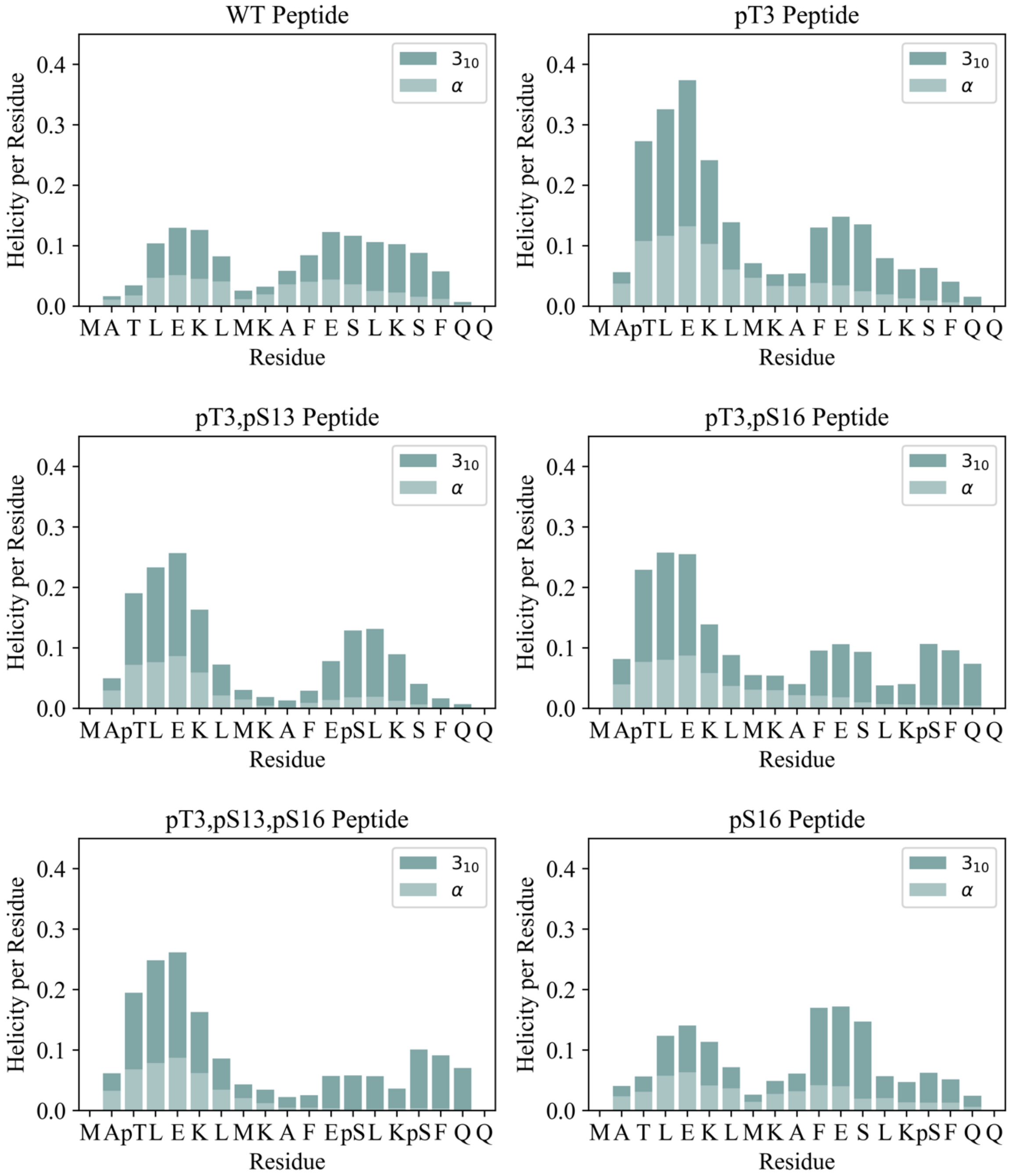

Supplementary Figure S5: Helicity per residue for the remaining peptides (not shown on Figure 2 in the main text).

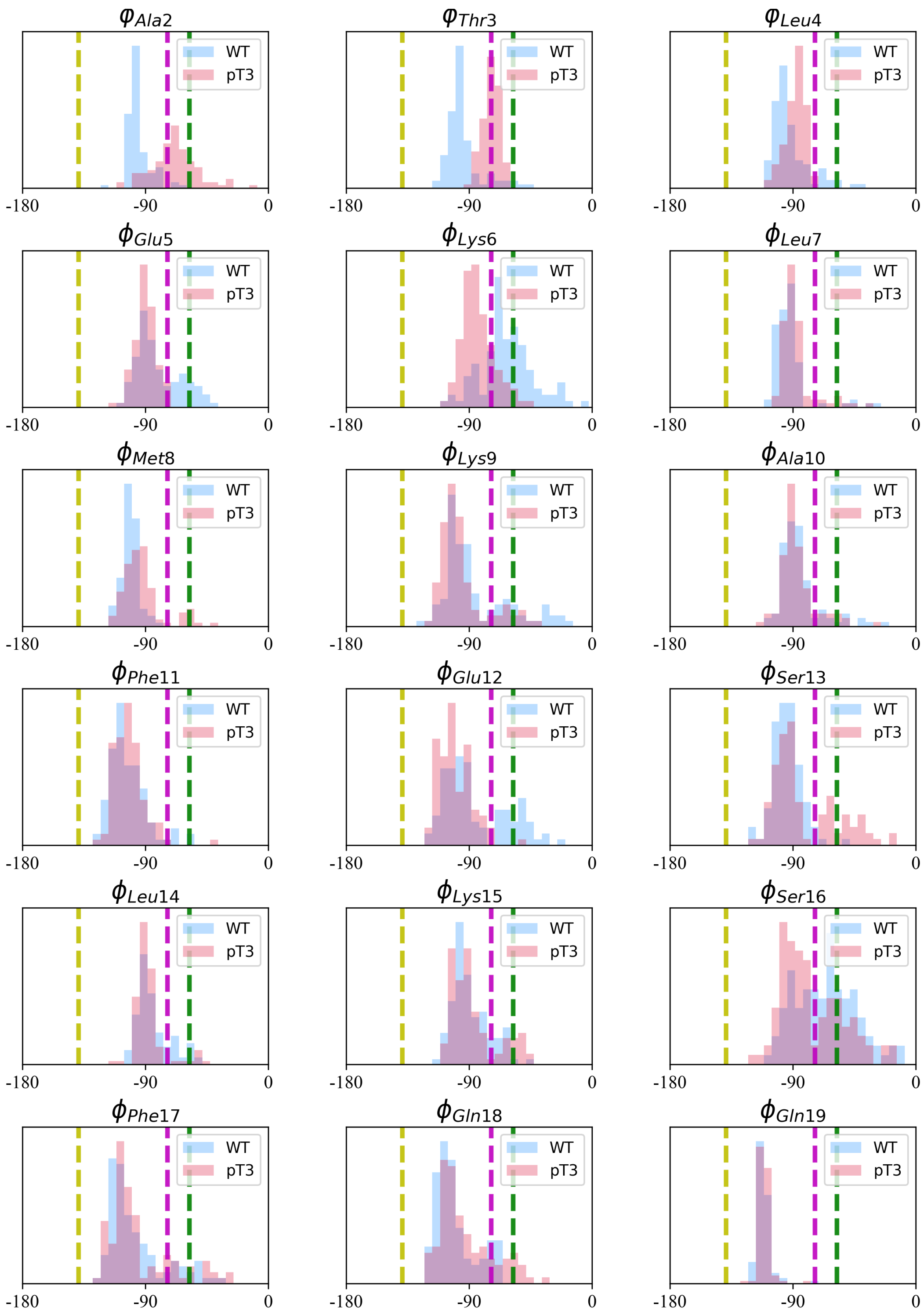

Supplementary Figure S6: Phi angles in WT vs pThr3 peptides showing that phosphorylation at Thr3 affects backbone dihedral angles of residues Lys6, Glu12, Ser13 and Ser16. Dashed lines indicate ideal phi angles for β-sheet (gold), 3_{10} helix (purple) and α-helix (green).

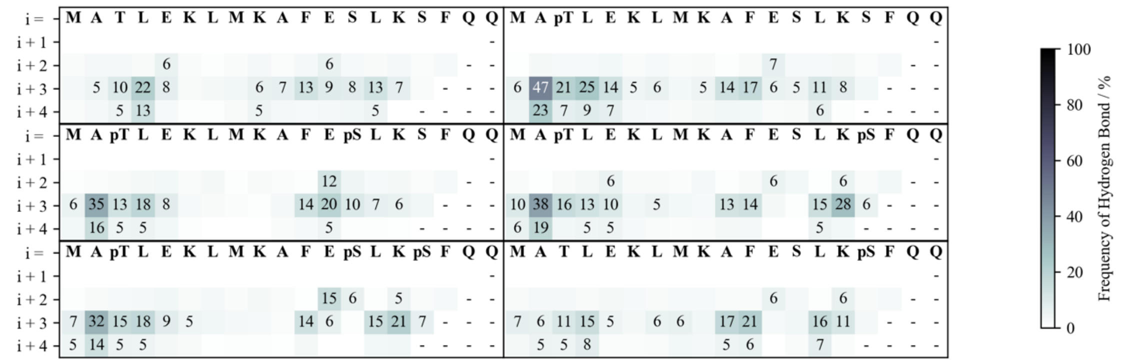

Supplementary Figure S7: Frequency of backbone hydrogen bond formation for the remaining peptides (not shown in Figure 3). The hydrogen bonds between the carbonyl of each residue (labelled in the 1st row) with the amide proton of the following four residues (rows) are shown.

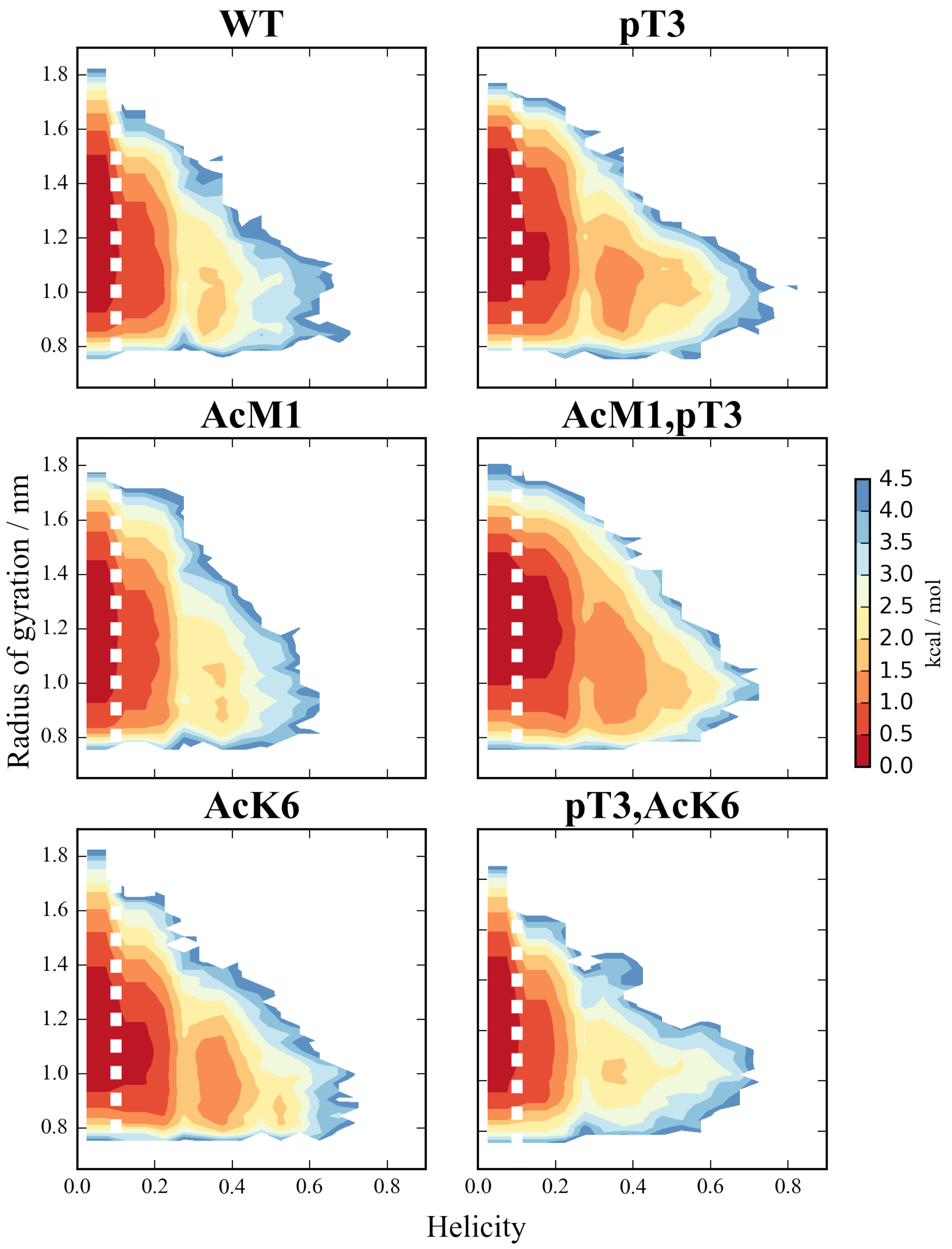

Supplementary Figure S8: Free Energy Surface of selected peptides based on radius of gyration and helical content.

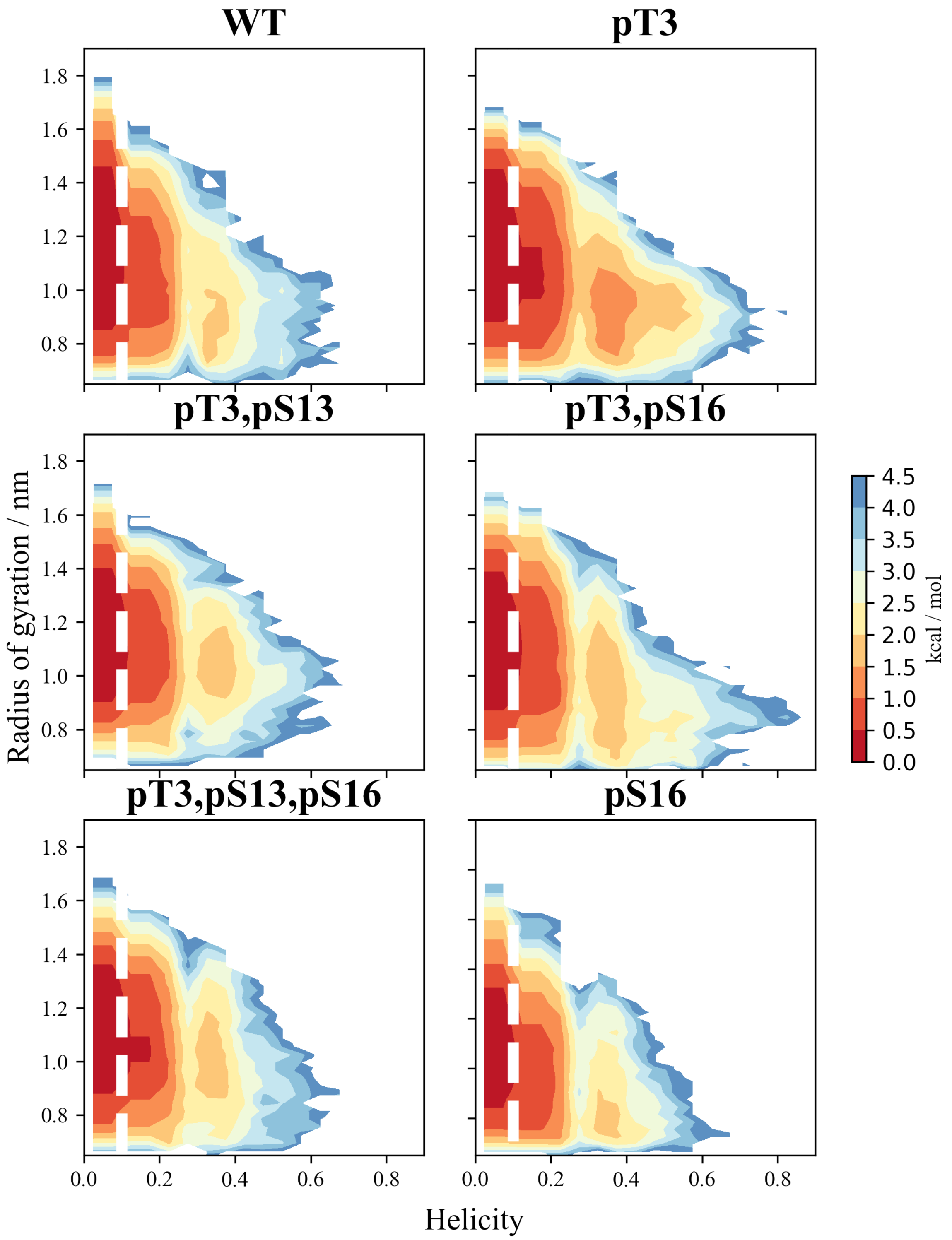

Supplementary Figure S9: Free Energy Surface of remaining peptides based on radius of gyration and helical content.

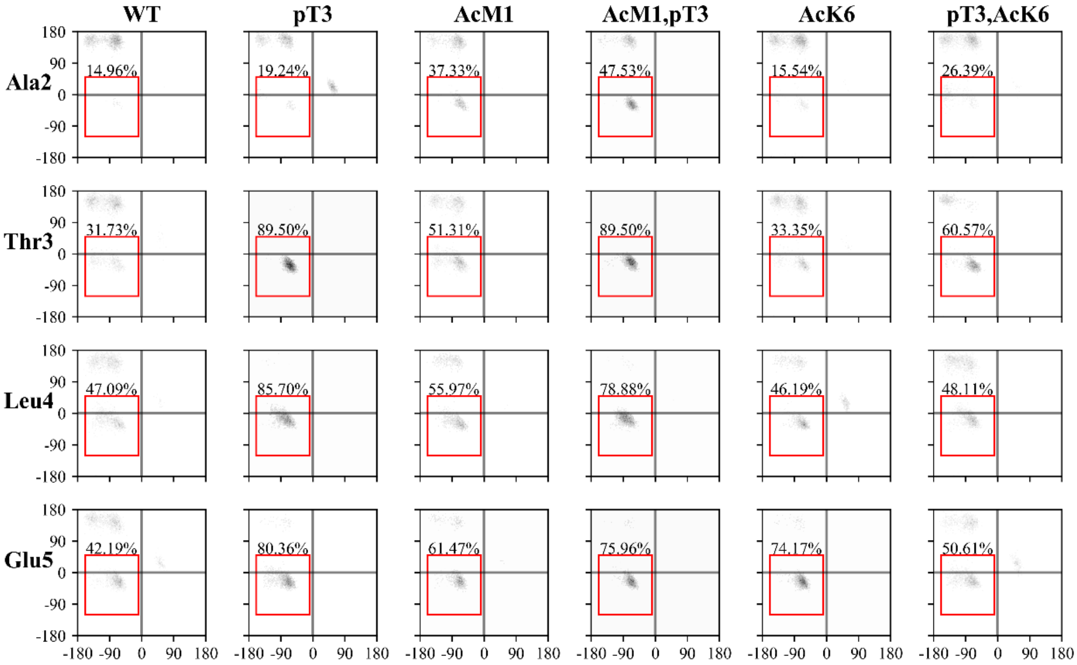

Supplementary Figure S10: Ramachandran plots of selected residues in WT, pThr3 and acetylated peptides, showing the fraction of alpha helical conformation.
